# The 3 levels of HIV stigma in the United States military: perspectives from service members living with HIV

**DOI:** 10.1186/s12889-021-11462-9

**Published:** 2021-07-15

**Authors:** Joseph M. Yabes, Phillip W. Schnarrs, Leroy B. Foster, Paul T. Scott, Jason F. Okulicz, Shilpa Hakre

**Affiliations:** 1 Infectious Disease Service, Brooke Army Medical Center, 3551 Roger Brooke Drive, JBSA Fort, Sam Houston, TX 78234 USA; 2 Department of Population Health, Dell Medical School, The University of Texas at Austin, Austin, TX USA; 3 U.S. Military HIV Research Program, Walter Reed Army Institute of Research, Silver Spring, MD USA; 4 Henry M. Jackson Foundation for the Advancement of Military Medicine, Inc., Bethesda, MD USA; 5 Emerging Infectious Diseases Branch, Walter Reed Army Institute of Research, Silver Spring, MD USA

**Keywords:** HIV, Stigma, Military, Sexuality

## Abstract

**Background:**

Epidemiological surveillance data indicate that a majority of HIV-infected in the United States (U.S.) military are African-Americans and men who have sex with men. There is limited research about barriers to HIV prevention among military service members and the unique factors that contribute to HIV stigma.

**Methods:**

A convenience sample of 30 U.S. service members were recruited from an infectious disease clinic. In depth interviews were conducted and data analyzed using a thematic coding process.

**Results:**

Two broad categories were identified: 1) Outcomes of HIV Stigma: Fear of Rejection, Shame, and Embarrassment; and 2) Strategies for combating stigma which include increasing HIV education and prevention resources. Military policies and institutional culture regarding sexuality were found to contribute to stigma.

**Conclusions:**

Participants identified a need for HIV education and suggested individuals living with HIV serve as mentors. A peer-to-peer intervention for delivering HIV prevention education may address these needs and reduce HIV stigma.

**Supplementary Information:**

The online version contains supplementary material available at 10.1186/s12889-021-11462-9.

## Background

In 2011, the United States Army Public Health Center (PHC, formerly Army Public Health Command) began monitoring HIV prevalence, incidence, and transmission risk, including sexual behaviors. Surveillance data from 2012 to 2014 indicates that African-Americans and men who have sex with men (MSM) are disproportionately affected by HIV in comparison to other racial or ethnic groups and heterosexual counterparts [1]. This initial report provides a detailed epidemiological profile of HIV in the United States (U.S.) Army and mirrors U.S. civilian data. However, it is limited by the lack of contextual findings in which to understand rates of HIV prevalence and incidence, as well as transmission risk [2].

Further, structural level factors can directly affect quality of mental health making HIV prevention efforts more difficult, as well as reducing engagement and retention in care among people living with HIV (PLWH) [3]. Literature has shown that structural level factors can negatively impact mental health quality of life which in turn decreased adherence to treatment plans [3]. The minority stress model describes the mechanism by which structural level factors are reproduced through individual level factors, contributing to experiences of poor mental health, and reduced engagement with HIV prevention and care [4, 5]. For example, a recent study by Garcia, Parker, Parker, et al., found that black MSM who lacked support from social networks engaged less with HIV prevention resources and tools like Pre-exposure Prophylaxis (PrEP) [6].

Garcia and colleagues’ research also demonstrates how stigma operates at a third level that involves the social norms associated with specific communities or social groups [6]. These meso-level factors contribute to stigma in community specific ways based on norms and beliefs associated with the group [6–8]. For example, the experiences of black MSM often include engagement, to some degree, with religious communities and beliefs that are anti-LGBTQ+. In this context, additional stigma exists regarding HIV and the sexual lives of MSM, for black MSM, especially given the importance of the church in black communities [8]. Meso-level factors point to the intersectional nature of stigma and the need to identify community specific determinants contributing to the construction of HIV-stigma [7].

There is limited research on the effects of HIV stigma on members of the U.S. military. Specifically, there is limited data on HIV related to community-specific factors contributing to HIV-stigma and the potential impact on HIV prevention efforts. The military is a unique context that requires investigation beyond our current understanding of HIV stigma in civilian populations. Practices unique to the military include the policy of mandatory HIV testing for all U.S. personnel prior to entry into service and biennially thereafter. A diagnosis of HIV precludes entry into service and may impact an existing member’s ability to deploy to certain military operations. These military policies regarding deployment and enlistment, as well as past policies regarding sexual minorities, and their sexual lives, such as Don’t Ask, Don’t Tell, shaped organizational cultural for decades, have the potential to impact career advancement, and continue to influence military culture [9]. These meso-level, or community specific factors, likely contribute to HIV stigma experienced by service members living with HIV, and those at risk for HIV, in several ways.

First, like other communicable diseases, PLWH are stigmatized because of their status. HIV negative individuals may have engrained beliefs about the lifestyle choices of PLWH such as sexual practices, orientation, promiscuity as well as regarding the transmission of HIV [10]. Second, PLWH are treated differently because of a poor understanding of current HIV treatment leading to fear of transmission [11]. Being perceived as gay, given the historical ties linking the HIV virus and the gay community in the United States, represents a third type of stigma related to heterosexism, that has implications for prevention, testing, and outreach in the military, as it does in civilian settings. Further, meso-level factors, such as current policies affecting deployment of service members living with HIV and those using PrEP for HIV prevention, as well as policies preventing PLWH with specialized skills from joining the military. Despite a medical system with universal HIV and PrEP coverage for active duty members this has the potential to negatively affect the health and wellbeing of those living with, and at risk for, HIV [12, 13]. Identifying and addressing meso-level factors is crucial for reducing HIV stigma experienced by populations associated who are members of social institutions, such as the military. This study sought to identify and describe barriers to HIV testing, prevention, and outreach, as well as the role HIV stigma plays in creating these barriers, from the perspective of members of the U.S. military living with HIV.

## Methods

### Sample and recruitment

The study was approved by two ethics committees, the Army Public Health Center’s Public Health Review Board (#14–311), and the Walter Reed Army Institute of Research’s Institutional Review Board (#1861E). Potential participants were recruited from a support group for PLWH at an infectious disease clinic in Texas while attending their medical appointment. Patients not involved with the support group were not approached for inclusion. Active duty service members with HIV infection were eligible for the study. Participation was voluntary and individuals were compensated $10 via a gift card for an online retailer. Interviews were performed from September 18, 2017 to May 8, 2018.

### Data collection

A total of 30 semi-structured, in-depth interviews were conducted by a retired U.S. Army service member who was trained by the lead author in interviewing techniques. Interview questions were developed through an iterative process, field testing the questions with members of the population of interest, and with input from the individual conducting the interviews after practicing his interview skills with members of the population of interest not part of the study [14]. Interview questions were developed specifically for this study and have not previously been published elsewhere. The interviewer met weekly with the lead author to discuss issues he encountered during the interview to determine if adjustments needed to be made (e.g. practice probing, strategies for building rapport, how to handle difficult or uncomfortable conversations because of the study topic) and to debrief and unpack the information from the interviews that week, as well as discuss any conversations, or interactions, which caused the interviewer to be uncomfortable. Interviews lasted between 45 and 60 min and were recorded using a digital audio recorder. Participants were asked 10 to 14 open-ended questions related to barriers to HIV testing and care, as well as how to reach service members who were at-risk for HIV. Additional File 1 displays the interview guide utilized in more detail. Interviews were transcribed and redacted to protect personally identifying health information.

### Data analysis

Redacted interviews were reviewed for accuracy and entered into a spreadsheet so that participant identification numbers were placed in the first column, research questions were placed in the first row, and each participant’s response was placed so that it was associated with the participant’s identification number and each question asked. Next data were analyzed using thematic coding. An iterative step-by-step thematic coding process was used to analyze the transcribed interviews [15, 16]. The lead author independently read each transcript several times to become familiar with the data. After reviewing the transcripts, open coding was done to generate an initial set of codes and associated definitions. All transcripts were analyzed again to ensure that codes were accurately applied. Similar codes were then grouped into broader themes and then themes were analyzed to identify theoretical connections. Codes and themes were discussed with other members of the research team.

### Researcher positionality

One of the co-first authors (PWS) independently analyzed all the interviews. PWS has been conducting research on HIV prevention and care, specifically in relation to multiple forms of stigma among sexual minority men for over a decade. Moreover, as a gay man PWS is aware of the role stigma plays in accessing HIV testing and prevention, but he has never been a member of the United States military and has limited experiences with conducting research in this context. This may have limited his understanding of the experiences of stigma among sexual minority men in the U.S. military. However, all other members of the study have worked in these contexts and provided feedback on initial codes. As a sexual health researcher with expertise in HIV stigma as it relates to testing and prevention, in a new context, this placed PWS in a position to identify themes related to HIV stigma in this new context.

## Results

Of the 30 individuals who participated interviews, the majority were members of the U. S. Air Force (80%, *n* = 24) and identified as male (97%, *n* = 29). Roughly half (47%, *n* = 14) were between 30 and 39 years of age and identified as African-American/black (57%, *n* = 17). Table 1 provides demographic characteristics of the sample.

**Table 1:** Participant Demographics

	n	%
Age (years)		
20–24	7	23
25–29	5	17
30–34	8	27
35–39	6	20
40–44	1	3
45–49	1	3
50–54	1	3
55–59	0	3
60–65	1	3
Sex		
Male	29	97
Female	1	3
Military Branch		
Air Force	24	80
Army	6	20
Race		
White	7	23
African-American/Black	17	57
Other	5	17
Unknown	1	3
Length of service at HIV diagnosis (years)		
1–5	12	40
6–10	4	13
11–15	7	23
16+	2	7
Unknown	5	17

### Qualitative themes

Three broad analytic categories were identified during analysis which included 1) individual level factors: Fear and shame; 2) Meso-Level Factors: Military policies and organizational history and culture, 3) Structural Level Factors: Addressing availability of education and sexual health resources, and social support for those who are at risk for, and living with, HIV.

### Individual level factors: fear and shame

#### Fear of HIV and HIV testing

When asked about barriers to HIV care and outreach, participants identified emotions that are associated with stigma. Interview participants discussed fear of HIV as an impediment to prevention and outreach. Participant 34 describes this fear relating it to getting sick or additional health complications, “In my opinion, they are, it’s more so, they are scared. You know? Just like I was when I definitely, when I started getting sick, I’m like, I’m not going to the doctor. You know?” he continues indicating that it was not only fear of HIV, but also fear of the additional complications:

What if they tell me this, what if they tell me that? And then … it was nausea. I already have sickness based on my other conditions. But it was just more to it, you know. And I'm like, well, yeah, let me really go. So, I didn't even go in to get checked for STDs. It was just a part of the process. And out of that process came the results, and when they told me I had to go see an infectious disease doctor, now you know you really get scared, like, "Okay, why am I having to go see this person? What's going on?" So it's more so, in my opinion, fear.

Participant 46 responded similarly, indicating that fear of “getting bad news” affects his decision to seek medical care, “Fear is probably [a barrier]. I mean that’s what stops me sometimes from being seen even like if it’s not HIV related like I hate going to the doctor because if I get bad news I’d rather have no news than bad news.” Further he explains that had he not received a diagnosis accidentally during a visit to an emergency room fear would have delayed his decision to seek out HIV testing:

I mean if I wouldn’t have gone to the ER when I found out, or what I was about to find out about the HIV, I probably wouldn’t have found out for months and who knows, things could have been worse, I could have spread it to more people, I could’ve caught it late on and my CD4 count have been lower, there’s all those factors. Yeah, exactly, that’s a big thing.

Similar to civilian populations, fear of HIV influenced decisions about HIV testing. This HIV testing avoidance is more explicitly described by participant 32:

Fear. They do not want to face their horrors. They're not wanting to face their fear like [a positive HIV result] might actually happen. You know what I'm saying? Because, I mean, honestly, for me, like, when I really thought about it, like, my body was going through subtle changes. You go to the doctor. But, you know, [the doctor] says you've got that black plague, you know, it's always got the worst thing ever.

Across interviews, fear of HIV was a factor that contributed to HIV testing avoidance. However, as participant 32 alludes to, by drawing a comparison between HIV and the black plague, there is also a fear related to being a social pariah.

### Fear of rejection

Fear of rejection is the second component of the broader theme of fear at the individual level. Participant 34 discussed fear of HIV not just in terms of his health, but also as being rejected by others, stating:

And then at the same time, which is something I, I had to come to truth with, just the rejection. You know? Well, if I find out, you know, is my parents still gonna like me or are they gonna kick me to the curb or my friends I hang out with all the time, are they gonna be like, "Ooh, I don’t want to touch you. Don’t want to be around you." You know? So just the rejection of others is another factor that I think personally played into the role of actually wanting to get tested and actually find out if you're negative or positive. It is a tough one.

This fear of rejection was not focused on sexual partners, but fear that his family and friends might not “like” him. Participant 35 suggests that fear associated with testing positive for HIV, and being labeled as the other, is a barrier:

Well definitely the stigma, I mean, is out there. It’s very real. And it’s just horrible and I mean nobody wants to come down with a diagnosis like HIV. I mean you feel stained and marked, you know, like why did you want to know, like that you have HIV? So I understand completely why people don’t want to get tested, I mean, because after you get tested it’s real, it’s there, it’s permanent.

In addition to fear of being rejected by family, friends, and, as participant 35 described, being marked, concern about being rejected by other service members emerged. Participant 39 describes his fear of being shunned by others in his unit when he was newly diagnosed:

For me at the time, if we want to talk about emotions, I wasn’t afraid for my life, more so I was more worried about what other people would start to think about me. Like if I would start to be shunned or avoided because I was diseased or whatever. So I felt the more people who find out, the more people who know is not okay with me. I wanted to try to keep the spread of the news to as few people as possible. So my unit commander, I could understand, but I didn’t want anyone else in my unit to know or to find out unless I gave them permission and consent.

While this was a concern for participant 39, overall the sentiment was services members were treated with respect by others in the military:

They’re so professional it’s like yeah, they respect me. But for sure, I’ve been there for three years so obviously the leadership has changed but as an airman there, starting out, they were extremely professional, extremely supportive.

### Shame and embarrassment

In addition to fear, participants reported that shame and embarrassment were barriers to HIV testing and outreach, and often associated these emotions with fear of rejection. Participant 30 reported that shame regarding sexuality is a barrier to HIV testing:

I feel like - shame. It’s definitely shame, you know. I mean when someone - when you think you have something and you’re afraid to go to the clinic and be seen, you know, there’s a barrier of shame that goes there. And if you don’t overcome that, you just feel like I’m good. You’ll sit right here, you’ll have a STD and just deal with it. You know? Because you’re afraid that someone can identify your sexual - your sexual life and make judgement against it.

Participant 69 described shame and embarrassment specifically being seen at an infectious disease clinic:

More than likely it’s a fear of like seeing someone they know here and them wondering about that and they’re going to assume hey, because I’m being seen for HIV, they must be here for the same thing and he’s going to know immediately.

Similarly, participant 45 indicated that shame and embarrassment were also barriers to HIV testing and prevention. However, unlike others in the study, participant 45 specifically indicates this is an issue for members of the black community:

I mean people are ashamed and embarrassed, not me, but I think, you know, people can be embarrassed. Especially like, I think in the black community for some reason a lot of them are embarrassed and ashamed.

### Meso-level factors: military policies and protocols

Meso-level factors included two themes that were interrelated. First, participants discussed former and current military polices that discriminated against PLWH in the military and the need for this to changed. Second, service members described how both former and current military policy have been shaped by the history and culture of the military as it specifically pertains to HIV, but also the synergistic effect of discriminatory policies against sexual and gender minorities, as well as sexuality, and how they have contributed to HIV stigma in the military.

#### Military policy and HIV

Stigma was reinforced through meso-level mechanisms via policies which, for example, affect service members living with HIV and their ability to deploy: limiting career advancement, and causing some service members to prematurely leave the military prior to reaching their greatest earning potential further demonstrating direct implications for service members living with HIV:

I can sit here all day and tell you HIV is not a big deal and I’m over it and I’m doing well and I’m healthy and I’ll probably outlive you and everybody else in this clinic, in a good way, and that it won’t mark my life but, you know, the reality is HIV has marked my life, especially professionally and we can talk here all day, it’s great, you’re a normal person everything but the policies and the guidelines are still outdated (Participant 35).

Above, Participant 35 indicates that HIV has “marked my life, especially professionally” he continues, questions why he cannot deploy, and brings to light an experience unique to the military that contributes to HIV stigma for service members:

I mean like why can I not deploy? Why cannot I go to every assignment? I mean if my condition is managed by just a one-pill a day regimen and I’ve been stable for nearly a decade with not having a single relapse after I started care, like why should I be treated differently than somebody who doesn’t have HIV?

These unique type of HIV stigma in U.S. military exists in addition to what PLWH would experience as civilians, the ability to deploy. Service members with HIV infection are deployable with limitations that vary by service branch, type of job, and deployed location. Limiting the ability to deploy after receiving a positive HIV diagnosis likely adds to the difficulty of receiving this diagnosis. Not only do individuals have to contend with daily discrimination, and other individual level factors, because of their positive HIV diagnosis, but they are now visibly different than other members of the military because of their inability to deploy. Members of the military with a positive HIV diagnosis are “othered” because of their HIV status and then again because they are prevented from engaging in an activity that is unique to their role as members of the military, deployment.

In addition, to policies affecting deployment status, policies around HIV-testing, and more broadly sexual health screening, created barriers to HIV prevention:

I had a one on one with my doctor when I found out I could just email her and told her I needed to be tested, but you should just be able to go get a test when you want to get a test, and not have questions asked. Because people will tell you, well you're not due for a year. I think military testing needs to be done every six months regardless of your sexual orientation. Done. Not this yearly crap (Participant 21).

This barrier related to the frequency of HIV testing and “not being due” for one is further discussed by participant 39:

[W]e just wanted to get tested normally, and there was our doctor who, he didn't really want to test people. I guess it was if he was saving money on his EPR [enlisted performance report], OPR [officer performance report] or saying like he saved the Air Force so much amount of money in testing. It's like well, you should only be tested for this and this if you think you have this or you should – you know, you got tested here but basically if you wanted to get tested for everything or whatever, he would just kind of have resistance to that…

#### Military protocols and accessing sexual healthcare

Participants described two primary factors related to military healthcare process that contributed to HIV-stigma, which included regularly having to engage with new providers making it difficult to develop a patient-provider relationship, and healthcare providers as gatekeepers to prevention and care. Service members indicated difficulty that building rapport with the healthcare providers hinder the ability to disclose their needs. Participant 30 points to this difficulty:

You always change. Always a new PCM [primary care manager] and you have to get to know them and develop a rapport and trust again with that person. So like being on civilian side with some of my people that have HIV, they was like I have a relationship with my doctor. You know, they say the doctor has been their same doctor for years, you know.

Participant 30 continues indicating this constant change not only hinders the ability of patients to develop trust, but it also affects care as well:

But to be in the military and to have constant rotation of new providers and stuff, it’s - It does. It does [hinder trust]. And it hinders care too. Because you’re hesitant to go - you’re hesitant to go to someone like for instance like if I was a guy, male, and I see another male provider I’ll be hesitant to talk to him about - because maybe I’m still trying to figure out my sexuality and stuff and maybe you’re afraid he might judge you and call - think weird things about you.

What participant 30 describes is a unique experience for individuals in the military. Civilians living with HIV are able to form relationships with their healthcare providers overtime, allowing for the development of trust. However, this participant indicates that same opportunity is not possible as a member of the armed services living with HIV. The inability to develop rapport with providers is further discussed by participant 54, but expressed as the lack of confidentiality:

Stigma and the hoops that you have to jump through to go get seen. Going to the TMC [Troop Medical Clinic]. Had I been able – and I think I can go to public health and just say hey I want to get tested for this reason or that reason but I think I’m supposed to go to TMC first, see primary care provider and then he’ll order labs and then I have to go to lab and then I go to public health and say...and they’ll give me my results. But the hoops that you have to jump through.

He further elaborates describing a scenario in which it is not just the primary care provider who presents a potential barrier to accessing HIV/STI testing, but also others:

I had an NCO [Non-Commissioned Officer] who thought he had something – he didn’t have anything but he didn’t go get seen because of the hoops that he had to jump through and I had to convince him, I had to like make a bunch of BS – and this is before my diagnosis as well – I had to make up a whole bunch of reasons like, you know, it’s going to fall off if you don’t get it checked out, just extreme stuff. But he was like I don’t want to go talk to captain so and so at TMC just to have to go see specialist so and so for them to draw blood and just to have to go to public health and see some civilian nurse, and that kept him from getting checked out.

This requirement of permission for HIV-testing reinforces negative emotions associated with HIV-stigma, so much so that participant 54 continues and indicates he would rather experience the symptoms of and STI rather than going through the process of being seen:

Me personally, as a...that’s what would keep me from getting checked out is just because I have to jump through a million hoops and I have to see a bunch of people and they also make it seem as though your command team has to be involved if you have anything at all so it’s like well, fine, I’ll just live with burning pee every day.

It is the lack of confidentiality between patient and provider, experienced in the civilian world, that creates additional barriers to accessing sexual health services such as HIV and STI testing.

### Structural level factors: access to sexual health resources and social support

Participants also identified structural barriers to HIV testing and outreach including education and awareness around sexual health, and the need for social support regardless of HIV status. Across interviews, participants described how structural barriers contributed to HIV stigma in the U.S. military and offered strategies for resolving them.

#### Sexual health resources

Limited awareness and availability of HIV prevention education and resources were consistently identified as an issue among participants. Across interviews, participants indicated that members of the U.S. military were in need of more sexual health courses and a broader diffusion of sexual health education and prevention tools. This need for increased awareness of HIV and broader implementation of sexual health education is illustrated by participant 39’s response:

The best way to reach service members. Awareness. I would say for the time, I mean right now when we have any type of town halls, meetings, commanders calls where we want to bring up public announcements to the masses in our units like to prevent or curb drunk driving or safety-related messages for like winter safety or summer safety, whatever, throw in other things like health concerns. You might be talking about suicide awareness or sexual harassment prevention, well what about STI awareness like sexually transmitted infections.

Participant 54, conveyed the same need regarding sexual health education for service members when first arriving for his Advanced Individual Training (i.e., the second component of basic training when recruits learn skills and specifics of their military occupational specialty):

When I first got to AIT [Advanced Individual Training] I had to take a finance class, I had to take a...you’ve got all these classes the Army said you have to take so I have to take a finance, I had to take like a home buyer thing and all this other stuff. Go ahead and just slide STD prevention in there and let the senior NCOs talk about it, say hey, use condoms or don’t have sex at all, don’t rape people, and stuff like that.

Similarly, participant 34 expressed a need for broader diffusion of the educational program required for service members who are living with HIV:

I think personally they need to spread the program they have here among the other bases as far as, bases that also treat on the Army, Navy, other Air Force folks…But at the same time I think they should spread this three-day course out to other bases. You're having one day. You see the doc. You go home. Well, that's not gonna help me. You know? I need to know what I need to be doing to make sure that I'm taking care of myself correctly and all that stuff so if there was any suggestion I would make … is get this course taken to other places because that's what's helping me personally.

The need for additional sexual health training was also discussed by participant 31, but he also alluded to the need for educating healthcare providers in addition to service members:

But to answer your question that the classes, they're doing it right. And, like I said so much in fact I'm concerned about my you know single gay airmen that probably if they haven't already are going to go to their PCM and they're not getting the same information that we get here. Yeah. That's why it's such a positive here. Because I'm like, wow, I get a lot that they're probably not going to get.

#### Social support

Participants identified the need for creating social support to address HIV stigma. Participant 44 describes the need for social support, recognizing his role in engaging newly diagnosed service members, and providing a human connection:

The stigma needs to go away that people have and I think that will help with relationships, which will help with people being able to find people more, to understand that with treatment this is like not having it pretty much. It really is. I think the support group is probably one of the most…the best thing that they could do here but, honestly, I know it has to be a…it’s not a required thing but it’s like yesterday we had two or three initials that didn’t stay in there…I guess they just didn’t…they didn’t want…that’s why I stay, it’s why I go to these so I can talk to someone newly diagnosed and let them know they’re going to be fine.

Further participant 21 points to the lack of human connection in the training individuals receive:

There's no human approach to it. I did it back in the days when I got in front of a crowd of people. I have experience to share. Got people emotionally involved. Made them understand about themselves. There's no human aspect to anything we do anymore. It's all a CBT (computer based training) and you click, click, click, click through it and it’s done. How is that – doesn't do anything. You need real testimony. You need real people in front saying this is what can happen to you, and this is why you need to do what you do. And then also flip and be like you can still do all of this too, regardless of if you have this. It's all about what we say in mental health, breaking the stigma.

This perceived lack of human connection was resolved for participant 57 when he came to the infectious disease clinic. Not only did he indicate feeling like it was a family reunion, but also reported that “there was no other environment” like the one that allowed HIV positive military member to engage with one another.

But I, I, I kind of look at this kind of like as a family reunion. Once a year I get to come here and, and I get to comingle with people that are receiving treatment for the exact same thing, going through similar instances, and in a lot of cases I get to educate people on stuff that I've gone through. This is my environment. There is no other environment that I'm aware of. Because it's so specific. Number one, it's the military aspect of it. Even if there was a civilian HIV outreach type environment or group, I think that it would be very specific towards civilian care, and not really specific to the military folks and we just – you know, and you know very well, we just have a different mentality. We need this here because it's military members or veterans. People who have that background in military, who understand the military and understand that we're just different, you know.

## Discussion

The repeal of Don’t Ask Don’t Tell (DADT) has allowed the U.S. Armed Forces to collect data on specific sexual behaviors associated with HIV transmission among MSM without potentially causing service members to be discharged from the military. There’s increasing evidence available post-DADT that African-Americans and MSM are disproportionately impacted by HIV. While this surveillance data provides needed information about the sexual health of those serving in U.S. military, the current study provides a first step in addressing how to improve HIV prevention and outreach by identifying population specific factors, and the underlying mechanisms, for the construction of HIV stigma in the military. Moreover, this work aids in our understanding of the role of minority stress for members of the U.S. military as it relates to HIV testing and prevention. Stigma and discrimination are well understood to be forms of minority stress. Overall, our findings suggest that participants felt stigmatized or feared being othered as a result of an HIV diagnosis. However, it was not just feeling stigmatized, but also discriminatory policies that treat military members living with HIV differently, as was as historical policies such as DADT, that continue to hinder things such as HIV prevention outreach and underutilization of sexual resources, especially among sexual minority men in the U.S. military.

Individual level factors such as fear and shame contribute to stigma in the military. While fear of rejection is commonly associated with an HIV diagnosis in the civilian world, in the military the fear is associated with interpersonal relationships that are both personal and professional. Participants were concerned with ensuring that individuals in their units did not find out. This fear of being rejected or treated different by their fellow service members raised concern for interviewees, however across interviews individuals consistently reported they were treated with respect. This suggests that these fears stem from structural causes rather than interpersonal interactions. Further, the inability of PLWH to deploy likely impacts one’s identity as a member of the U.S. military, but also has implications for how HIV is perceived within this institution. In this context, HIV is associated not only with disease and sex between men, but also as an additional deficit specific to military service, the inability to deploy likely affecting how service members living with HIV believe others members of the military might perceive them in light of this limitation. These structural factors: limitations on deployment, training, accession, were created with the intention of serving the best interests of the military. However, these same factors may have unintended detrimental consequences and result in an increased attrition of already trained/indoctrinated personnel. Brundage and colleagues evaluated the length of military service following incident HIV diagnosis and found that while the duration of continued service has increased over time, at least 25% of service members separate from the military within 16 months following diagnosis [17]. Whereas previously lack of effective antiretroviral therapy and medical discharges could explain separation from military service it is likely that stigma continues to play a significant role in continued employment in the U.S. military. Changes in military policy may not be feasible but addressing the stigma associated with the inability to deploy because of HIV could reduce fear and shame related to HIV testing, outreach, and prevention. Furthermore, addressing these stigmas may also serve the armed forces from losing service members who would have otherwise remained on active duty service.

Much like the civilian world, fear and shame are barriers to HIV prevention and testing. The novelty here is the role that military policy and protocols play in the delivery of HIV prevention and testing services. The inability to build trust and perceived lack of confidentiality had real implication for HIV testing avoidance and also contribute to, and are reinforced by, individual level factors of fear and shame. This has broader implications for HIV prevention beyond just screening, but also for the delivery and uptake of pre-exposure prophylaxis for service members at risk for HIV.

Military personnel living with HIV are directly affected by meso-level factors such as military policies. One such policy is Army Regulation (AR) 600–110 which provides rules and guidance for PLWH in the U.S. Army [18]. AR 600–110 prohibits service members living with HIV from deploying and from being assigned overseas, which limits their ability for career advancement [18]. However, initiatives are in progress to provide waivers for select assignments and deployments in the Army. Policy regarding overseas deployment and assignments for the Air Force, Navy, and Marine Corps are somewhat more flexible. For example, the naval policy states that PLWH “may be assigned to selected ships and Outside the contiguous United States (OCONUS) commands as agreed on by all three consultants and the receiving command; the receiving command has the final say on acceptance [19].” The Air Force takes a similar stance allowing case by case review for overseas assignments. Despite these more inclusive policies they still may limit career advancement for members with HIV infection. Additionally all services bar individuals with HIV from enlisting preventing PLWH, with specialized skills, from enlisting in the U.S. Armed Forces even though medication could be made readily available [18–20]. These same policies create a culture that promotes fear and shame at the individual level.

Across interviews, there was a perceived lack of awareness of HIV prevention that respondents felt could be addressed by making sexual health education provided to service members living with HIV more broadly available to all members of the military. The armed forces require annual face to face training on sexual assault, however more comprehensive sexual health training modules that incorporate STI/HIV are not mandatory and only available through service specific public health websites. The answers from respondents involved with this study suggest that these resources are underutilized. Previous investigations of STIs in the military support an underutilization of sexual health resources [21]. Military populations have shown increasing rates of STIs analogous to the civilian population, however the military at times has exhibited even higher rates of STIs such as during deployment [22]. A previously proposed explanation for high rates of STIs in the military, at least in part, has been the increased screening policies. Current requirements include annual well-woman examinations for active duty women, females under the age of 25 years undergo yearly chlamydia nucleic acid amplification test evaluation, and HIV screening is to be conducted: prior to deployment, when transitioning to overseas assignments, on a biannual basis, upon entry to drug or alcohol treatment programs, prior to incarceration, and for clinically indicated reasons [18–21]. The current study instead sheds new light on a lack of awareness that sexual health resources exist outside of those that are required. Increased advertisement of these resources aimed at overall sexual health and HIV prevention would not only impact public health outcomes for the military population as a whole but also reduce HIV stigma by moving the conversation away from a medical model towards a more comprehensive conversation about sexual wellness.

Effective strategies for reducing HIV stigma include improving social support for and engagement with PLWH. Across interviews, participants described a lack of social support for PLWH in the military. However, there was a strong desire from participants to be resources for those who are newly diagnosed with HIV. Similarly, service members living with HIV could also be a resource for those who are HIV negative. Training HIV positive service members to deliver HIV education would address multiple issues associated with HIV stigma. It could also serve to fill a knowledge gap. A survey aimed at understanding Air Force primary care provider’s knowledge and practices in regards to MSM medical care of was conducted with 39% of respondents reporting they had never received training in the medical care of MSM [23]. With the MSM population disproportionately affected, it is likely that this lack of knowledge directly impacts the care of PLWH and almost certainly with HIV prevention efforts. Similarly, a separate survey regarding PrEP usage among tri-service military health care providers showed that 49% of respondents rated their knowledge of PrEP as poor [24]. Utilization of a peer-to-peer strategy would serve to fill this education gap regarding HIV prevention and care for the MSM community by taking advantage of a motivated pool of trainers to educate the patients themselves who could then act as their own advocates. It also has the potential to increase awareness of HIV and HIV prevention, reduce stigma through engagement with those living with HIV, and increase social support through the sharing of experiences from those living with HIV and serving in the military.

There are several limitations to this study. First the study included a small sample size and the study participants were limited to a convenience sample with a limited scope for recruitment. These factors may make it difficult to extrapolate the data to a broad range of patients. Similarly while the military clinic where the study was conducted provided medical care to members of all the armed forces, due to geographic differences in military base locations the population was overwhelming represented by the Army. It is possible that HIV positive members of different military services, with their different cultures, traditions, and customs may have different perceptions and experiences. Another limitation was that the semi-structured interviews did not include directed questions to assess whether any of the participants ever sought care outside of the military health system. Furthermore, it is possible that HIV positive service members found such a degree of stigma within the military health system that they did in fact receive their care in the civilian sector and would not have been included within our study. Finally, having a single individual analyze the qualitative results may be a source of bias in the interpretation of the data. The strengths of this study include its utilization of semi-structured interviews purposely utilized to allow for maximum participant input and a broad capture of information. The use of single interviewer is another strength that helped to ensure that all participants had a similar experience.

## Conclusion

Current military policies related to HIV and institutional culture contributed to the structural and psychosocial barriers testing, prevention, and outreach. From the perspective of those living with HIV in the U.S. military, there is a need for broader diffusion of HIV education. Our findings suggest that those living with HIV are willing to be advocates and facilitators of groups for HIV individuals who are newly diagnosed as well as those who are risk for HIV. Given the unique setting, a peer-to-peer intervention for delivering HIV prevention education and increasing knowledge of prevention resources may be well suited for addressing multiple factors contributing to HIV stigma.

## Supplementary Information

**Additional file 1.** Qualitative Script HIV Stigma in the US Military. Interview script developed and utilized for the study above.

## Data Availability

Due to the sensitive nature and higher likelihood of identification of patients, which could realistically include adverse impact to HIV-infected United States Army personnel still in service and possible insurability and employability impacts, data cannot be made publicly available. For questions about data, contact the Walter Reed Army Institute of Research Public Affairs Office at usarmy.detrick.medcom-wrair.mbx.public-affairs@mail.mil.

## Abbreviations

MSM: Men who have sex with men
U.S.: United States
PLWH: People living with HIV
PrEP: Pre-exposure prophylaxis
EPR: Enlisted performance report
OPR: Officer performance report
PCM: Primary care manager
TMC: Troop medical clinic
NCO: Non-commissioned officer
AIT: Advanced individual training
DADT: Don’t ask don’t tell
OCONUS: Outside the contiguous United States
