# Supplementary material for: The 3 levels of HIV stigma in the United States military: perspectives from service members living with HIV
Source: BMC Public Health. 2021 Jul 15;21:1399. doi: 10.1186/s12889-021-11462-9 (PMC8281656; doi:10.1186/s12889-021-11462-9)
Supplement: Supplementary file 1 — Additional file 1. Qualitative Script HIV Stigma in the US Military. Interview script developed and utilized for the study above. [file 12889_2021_11462_MOESM1_ESM.docx]

Qualitative Script HIV Stigma in the US Military

INTRO

*My name is…..What may I call you?*

*Do I have your permission to record this session?*

EXPLAIN INTERVIEW

*Please be aware some of these questions can be personal in nature. At anytime please feel free to stop. You can stop at anytime and always feel free to be brutally honest with me.*

WARNING UP FRONT

*There may be some pauses for notes while we are going through these questions. Again, some of these questions are personal in nature. Additionally some of these questions may sound repetitive and/or redundant.*

- *How are you doing?*
- *Tell me about your experience leading up to your diagnosis/leading up to your first visit here.*
- *How were you treated?* (Be encouraging, only 1 in 5 know they are HIV positive)
- *How did you become comfortable with your primary care provider in discussing:*
  - *Sex life*
  - *Sexuality*
  - *STIs/HIV/sexual health needs*

FOLLOW UP

- *Why did it make you feel comfortable?* (elaborate and compare…i.e would you say?)
- *What specific thing did your provider do to make you feel comfortable talking about your sex life, sexuality, STIs/HIV/sexual health needs*
- *What specific thing(s) did your provider do to make you feel comfortable talking about a new sti or your sex life/sexual health needs? Why did this make you feel comfortable?*
- *What specific thing(s) make you feel (un)comfortable talking about sex life, sexuality, STIs/HIV/sexual health needs and why?*

SCENARIO

- *YOU are now the provider for this clinic. How would you make your HIV positive clients comfortable talking about HIV and STI’s? With sexuality? With sex life? With you? Why would you do these things?*

FOLLOW UP

- *How did you come to trust your provider? Can you give me examples?*
- *How did you come to trust your provider when talking about:*
  - *Sex life*
  - *Sexuality*
  - *STIs/HIV/sexual health needs*
- *Can you give me examples?*
- *And how did that work?*
- *What things did your provider do to make you distrust them when talking about your:*
  - *Sex life*
  - *Sexuality*
  - *STIs/HIV/sexual health needs*
- *And how did that work?*
- *What barriers to sexual health screening do think exist? Or what things do you see stop someone from being seen?*
- *What is the best way to reach service members who have a relationship with /dealing with HIV?*
- *What’s working well with your visits here & what’s not working well?*
- *In case we have any questions about what you stated or need clarification, is it okay that we contact you?*

.
